# A Retrospective Study on the Histological and Clinical Features of 92 Feline Oral Neoplasms in Thailand

**DOI:** 10.3390/ani16050717

**Published:** 2026-02-25

**Authors:** Pitak Anusorn, Supreeya Srisampane, Charuwan Wongsali, Pollawat Jariyarangsrirattana, Chakkarin Satthathum, Naris Thengchaisri

**Affiliations:** 1 Surgery Unit, Kasetsart University Veterinary Teaching Hospital, Faculty of Veterinary Medicine, Kasetsart University, Bangkok 10900, Thailand; docboxbox@gmail.com (P.A.); fvetpwj@ku.ac.th (P.J.); fvetcrs@ku.ac.th (C.S.); 2 Veterinary Diagnostic Center, Faculty of Veterinary Medicine, Kasetsart University, Bangkok 10900, Thailand; yyungvet@gmail.com (S.S.); charuwan6@gmail.com (C.W.); 3 Department of Companion Animal Clinical Sciences, Faculty of Veterinary Medicine, Kasetsart University, Bangkok 10900, Thailand

**Keywords:** cat, oral neoplasm, histopathological, neoplasm origin, clinical behavior

## Abstract

This retrospective study evaluated 92 feline oral neoplasms diagnosed in Thailand to characterize neoplasm types, anatomical distribution, and clinically relevant associations. Epithelial neoplasms accounted for 73% of all neoplasms, with squamous cell carcinoma being the most common (67%). Other epithelial neoplasms such as ameloblastoma, amelanotic melanoma, and adenocarcinoma were uncommon. Mesenchymal neoplasms (27%) were more heterogeneous, encompassing fibrosarcoma, osteosarcoma, lymphoma, plasma cell tumors, and several rare entities. Cats with epithelial neoplasms were significantly older than those with mesenchymal neoplasms (11.0 ± 3.8 vs. 6.8 ± 4.1 years old; *p* < 0.001), while body weight, sex, clinical stage, and perioperative mortality did not differ between neoplasm types. Breed distribution varied by neoplasm origin, with epithelial neoplasms more common in domestic shorthair cats, and the maxillary gingiva was the most frequently affected site. Neoplasm size strongly correlated with disease stage, as neoplasms > 2 cm were associated with advanced stages, while neoplasms ≤ 2 cm were typically stage I. Antibiotic use was significantly more frequent in senior cats compared to mature/adult cats (*p* = 0.014). In summary, SCC is the most common oral tumor in cats, indicating the importance of routine oral examinations, particularly in senior cats.

## Simple Summary

Oral neoplasms are a common and serious problem in older cats but are often difficult to recognize early. We reviewed 92 feline oral neoplasms diagnosed in Thailand and found that most were epithelial in origin, with squamous cell carcinoma being the most frequent. Cats with epithelial neoplasms were significantly older than those with mesenchymal neoplasms. The maxillary gingiva was the most commonly affected site, and neoplasm size showed a strong correlation with disease stage, as neoplasms larger than 2 cm were typically associated with advanced stages. These findings emphasize the importance of routine oral examinations in senior cats, as early detection may improve staging accuracy and expand treatment options.

## 1. Introduction

Feline oral neoplasms represent a diverse group of lesions, encompassing both benign and malignant types [1,2,3,4]. Among malignant forms, feline oral squamous cell carcinoma (OSCC) is commonly observed; in this study, “SCC” refers specifically to feline OSCC. Clinical signs of SCC can vary according to neoplasm location [5]. Cats with oral neoplasms often present with halitosis, excessive salivation, difficulty eating, oral bleeding, or anorexia [6,7,8]. Histopathological evaluation allows differentiation of malignant and benign lesions [1,4] as well as neoplasm classification according to cell origin as epithelial, mesenchymal, or neuroectodermal [9]. The frequency and type of feline oral neoplasms have been documented in epidemiological surveys from different geographical areas. In Portugal, SCC was the most frequently diagnosed oral neoplasm, with most cases classified as malignant [3]. Similarly, in Switzerland, SCC predominated among malignant oral neoplasms, followed by fibrosarcoma (FSA), melanoma, and adenocarcinoma [4]. In Korea, a retrospective study found SCC to be the most common malignant oral neoplasm, while peripheral odontogenic fibroma (POF) was the most frequent benign lesion [10]. Collectively, these studies indicate that SCC is the primary malignant oral neoplasm in cats from these regions [3,4,10].

Advanced age is associated with the occurrence of oral neoplasms in cats, although sex does not appear to be a consistent factor [2,4]. Most cases involved cats of non-specific breeds [2]; however, European Shorthairs were overrepresented in Portugal and Switzerland [3,4], and domestic shorthair (DSH) cats accounted for more than half of reported cases in another study [11]. Neoplasm location in the oral cavity varies by neoplasm type. In cats with SCC, lesions have been reported in the tongue or sublingual region [4] and in the gingiva [3]. Importantly, true glossal SCC is anatomically distinct from sublingual SCC, and these entities should not be considered synonymous.

For benign neoplasms, POF was mainly located in the caudal region [4]. In a small case series, most feline oral melanoma occurred in the maxilla [7], although neoplasms have also been reported in the lips, buccal mucosa, palate, and other jaw regions [8], suggesting an anatomical predilection by neoplasm types. Previous studies have also suggested that environmental exposures may play a role in SCC development [12,13,14,15,16]. Ultraviolet (UV) radiation has been shown to induce mutagenic DNA photoproducts in the skin, a mechanism well characterized in carcinogenesis models [17]; however, its role in the development of oral SCC remains uncertain. Geographic and environmental factors may influence the relative distribution of feline oral neoplasm types, and although Thailand’s tropical climate is characterized by year-round sunlight exposure, whether this may contribute to regional variation in prevalence remains unknown.

This study, therefore, aimed to improve the geographic understanding of feline oral neoplasms by retrospectively analyzing cases from Thailand, focusing on histological origin, anatomical distribution, and associated clinicopathologic features. Inclusion of cases from Southeast Asia provides region-specific data for feline oral neoplasm.

## 2. Materials and Methods

### 2.1. Study Design and Ethical Approval

This retrospective study reviewed feline oral neoplasm cases presented to the Kasetsart University Veterinary Teaching Hospital, Faculty of Veterinary Medicine, Kasetsart University, Bangkok, Thailand, between January 2018 and December 2024. Ethical approval was obtained from the Kasetsart University Institutional Animal Care and Use Committee (approval number ACKU69-VET-005) and the Ethical Review Board of the Office of the National Research Council of Thailand (NRCT license U1-07639-2561).

### 2.2. Study Population

**Figure 1:**
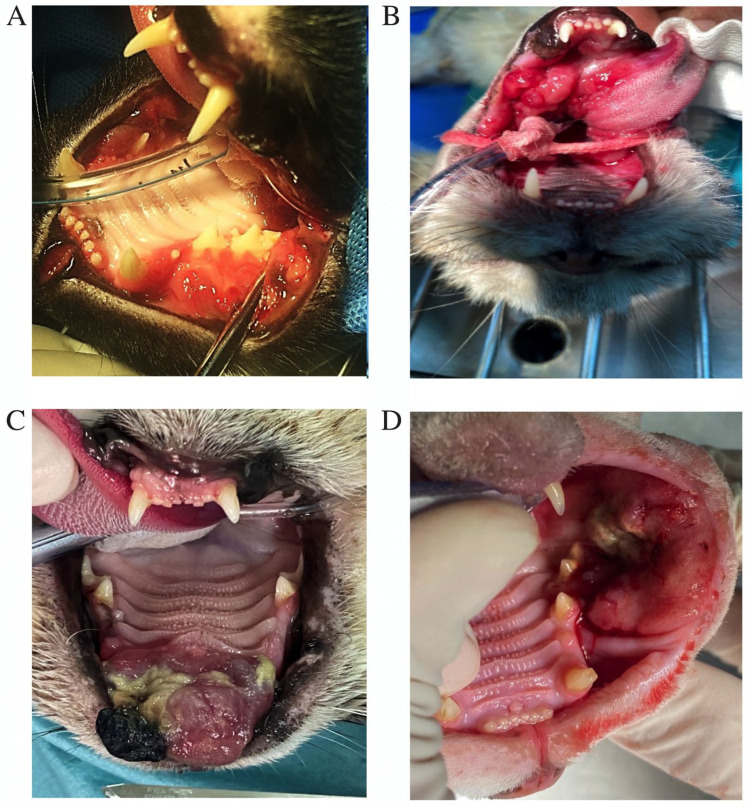
Representative gross appearances of commonly encountered feline oral neoplasms: (**A**) SCC-like lesions appear as ulcerated, irregular, and invasive masses on the maxillary gingiva; (**B**) SCC in the sublingual–lingual region; (**C**) oral FSA is shown as a firm mass and an ulcerated lesion on the rostral hard palate; and (**D**) malignant melanoma presents as a variably pigmented, ulcerated buccal mass adjacent to the premolar teeth.

A total of 92 cats with histologically confirmed oral neoplasms were included. Cases were excluded if clinical information was incomplete or if the histopathological diagnosis was inconclusive. All tissue samples were examined and diagnosed by licensed veterinarians with Diplomate status from the Thai Board of Clinical Pathology (DTBCP) and professional experience in veterinary diagnostic pathology. Clinical records were reviewed to extract data on age, sex, breed, body weight, body condition score, feeding type, neoplasm location and size, histological classification, clinical stage, prior use of antibiotics or corticosteroids, presence of concurrent dental disease, and perioperative outcome. Age categories were defined according to the American Association of Feline Practitioners (AAFP) life-stage guidelines for domestic cats [18]. Representative gross appearances of the oral neoplasms evaluated in this study are presented in Figure 1 for illustrative purposes.

### 2.3. Neoplasm Staging and Classification

Neoplasms were staged using the World Health Organization (WHO) Tumor–Node–Metastasis (TNM) system adapted for domestic animals [19]. Stage I included neoplasms < 2 cm without regional lymph node involvement; Stage II comprised neoplasms 2–4 cm without nodal involvement; Stage III encompassed neoplasms > 4 cm or any size with regional lymphatic involvement; and Stage IV indicated distant metastasis. For statistical analysis, neoplasms were also categorized by size as ≤2 cm or >2 cm. Because tumor size is an integral component of the TNM staging criteria, no inferential analysis was conducted to assess an association between size category and stage, thereby avoiding circular interpretation. Due to substantial loss to follow-up in this retrospective cohort, survival data were not consistently available. Accordingly, staging was recorded to describe neoplasm burden at the time of diagnosis rather than to infer prognosis or survival outcomes, and was included to provide clinical context only.

### 2.4. Histopathological Classification

Tissue samples were fixed in 10% neutral buffered formalin, routinely processed, and stained with hematoxylin and eosin (H&E). All lesions were classified by neoplasm origin as epithelial, mesenchymal, or neuroectodermal [9]. For analysis, neoplasms were grouped as epithelial or mesenchymal. Representative epithelial neoplasms included SCC, adenocarcinoma, and ameloblastoma [20], while mesenchymal neoplasms included FSA, osteosarcoma (OSA), chondrosarcoma, rhabdomyosarcoma, fibrolipoma, POF, and round cell neoplasms such as lymphoma, plasma cell tumor (PCT), and mast cell tumor (MCT) [6,20]. Although melanomas arise from neural crest cells, neoplasms with epithelioid morphology were classified as epithelial for statistical purposes [21]. For each neoplasm type, the defining histomorphologic features forming the basis of classification are described below.

**Figure 2:**
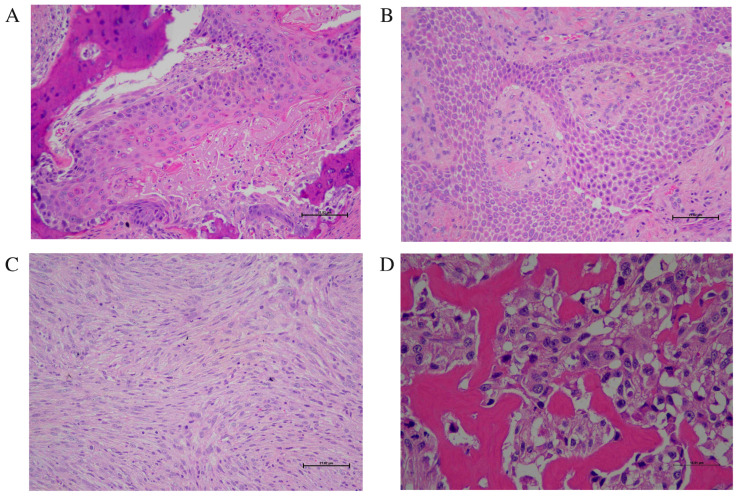
Representative histopathological features of feline oral neoplasms: (**A**) SCC: nests of atypical squamous epithelial cells with keratin pearl formation and invasion to bone trabeculae. The neoplasm was composed of large cuboidal to polygonal squamoid cells with nuclear atypia. (H&E); (**B**) Ameloblastoma: an uncapsulated infiltrative epithelial neoplasm with peripheral palisading present in many islands containing loosely arranged, stellate reticulum-like cells supported by fibrous stroma. The columnar cells with hyperchromatic nuclei at the basal layer exhibit peripheral palisading with reverse polarization away from basement membrane (H&E); (**C**) Undifferentiated sarcoma (possible fibrosarcoma): spindle-shaped neoplastic cells in interlacing bundles; Melan-A negative (IHC); and (**D**) OSA: malignant osteoid production by pleomorphic spindle cells with thin trabeculae of neoplastic osteoid eosinophilic, homogenous, glassy with irregular contours and osteoblastic rimming growing around trabeculae, with necrosis and bizarre giant cells in the stroma; neoplasm cells were spindly, oval or round of variable size, with osteoclast-like multinucleated giant cells (H&E).

SCC was diagnosed based on invasive proliferation of atypical squamous epithelial cells exhibiting keratinization and variable degrees of cellular atypia and mitotic activity (Figure 2). Adenocarcinoma was defined by malignant glandular epithelial proliferation with infiltrative growth. Ameloblastoma was identified by odontogenic epithelial islands with peripheral palisading and reverse nuclear polarization.

Amelanotic melanoma was diagnosed in non-pigmented malignant neoplasms in which melanoma was included in the differential diagnosis based on morphology and confirmed by positive cytoplasmic immunoreactivity for Melan-A in neoplastic cells.

Spindle cell sarcomas were diagnosed according to characteristic histomorphologic features and matrix production. FSA was identified as a malignant spindle cell neoplasm arranged in interlacing bundles with cellular atypia and mitotic activity. OSA was defined by malignant mesenchymal cells producing osteoid matrix. Chondrosarcoma was characterized by malignant chondrocytes embedded within a cartilaginous matrix exhibiting cellular atypia and infiltrative growth. Rhabdomyosarcoma was diagnosed as a poorly demarcated mesenchymal neoplasm composed of round to spindle-shaped cells arranged in clusters and nests, demonstrating differentiation toward myotubes, including strap cells and multinucleated forms with identifiable myofibrils. When present, cross-striations supported skeletal muscle differentiation. Tumors lacking definitive differentiation were classified as sarcoma, not otherwise specified (NOS).

Round cell neoplasms were diagnosed based on cytomorphology and growth pattern. Lymphoma was identified by diffuse infiltration of poorly circumscribed, unencapsulated sheets of monomorphic round cells effacing normal tissue architecture. Neoplastic cells exhibited round hyperchromatic nuclei with granular chromatin, multiple nucleoli, scant cytoplasm, anisocytosis, and high mitotic activity, consistent with lymphoid origin. MCT was confirmed by diffuse infiltration of neoplastic mast cells and demonstration of cytoplasmic metachromatic granules using toluidine blue staining. PCT was diagnosed based on characteristic plasmacytoid morphology, including eccentric nuclei, abundant eosinophilic cytoplasm, and a perinuclear clear zone.

Benign mesenchymal proliferations were classified based on differentiation and absence of cytologic atypia. Fibrolipoma was diagnosed as a well-differentiated mesenchymal neoplasm composed of mature adipocytes admixed with abundant fibrous connective tissue, lacking significant cellular atypia or increased mitotic activity. POF (also termed fibromatous epulis of periodontal ligament origin) was diagnosed as a gingival-based fibrous proliferation composed of densely cellular spindle to stellate mesenchymal cells within a collagenous stroma resembling periodontal ligament tissue. Variably sized islands of cemento-osseous matrix were present, and the lesion was covered by hyperplastic stratified squamous epithelium with elongation of rete pegs. Cellular atypia was minimal, and mitotic figures were rare or absent. Immunohistochemistry was performed on formalin-fixed, paraffin-embedded sections using standard diagnostic laboratory protocols. Appropriate positive control tissues were included for each antibody, and negative controls were prepared by omission of the primary antibody.

Immunohistochemistry was applied selectively in cases requiring lineage clarification. Melan-A immunohistochemistry was performed when melanoma was included in the differential diagnosis. Toluidine blue staining was used to confirm MCT. Lymphoma and PCT were diagnosed primarily based on characteristic morphology; immunophenotypic subclassification was not performed due to limitations inherent to the retrospective design. Mesenchymal sarcomas were diagnosed based on histomorphology and matrix production; vimentin or skeletal muscle marker immunostaining was not routinely performed. Tumor grading and mitotic index quantification were not uniformly available and were therefore not included in the analysis. Because this was a retrospective study, ancillary diagnostic procedures were limited to those performed at the time of original case submission and were not uniformly applied across all cases. Representative histopathologic features of epithelial and mesenchymal neoplasms are shown in Figure 2.

### 2.5. Statistical Analysis

Continuous variables (age, body weight) are presented as mean ± standard deviation (SD) and compared using Student’s *t*-test. Categorical variables (sex, breed, clinical stage, medication use, neoplasm location) were analyzed with the Chi-square or Fisher’s exact test, as appropriate. Statistical analyses were performed using Stata version 12.1 (StataCorp, College Station, TX, USA). Statistical significance was defined as *p* < 0.05.

## 3. Results

**Figure 3:**
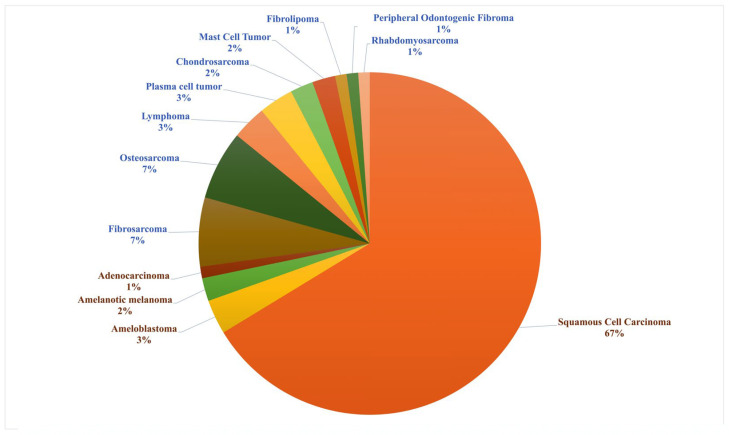
Histological distribution of feline oral neoplasms in 92 cases from Thailand. Epithelial neoplasms include squamous cell carcinoma, ameloblastoma, amelanotic melanoma, and adenocarcinoma. Mesenchymal neoplasms include fibrosarcoma, osteosarcoma, lymphoma, plasma cell tumor, chondrosarcoma, mast cell tumor, fibrolipoma, peripheral odontogenic fibroma, and rhabdomyosarcoma.

Among the 92 cats diagnosed with oral neoplasms, epithelial neoplasms were the predominant histologic category (Figure 3). These included SCC, ameloblastoma, adenocarcinoma, and melanoma (amelanotic), with SCC representing the majority (61 cats, 67%). Other epithelial neoplasms were less frequent: ameloblastoma (3%), amelanotic melanoma (2%), and adenocarcinoma (1%). Mesenchymal neoplasms displayed greater histological diversity, comprising FSA, OSA, chondrosarcoma, and round cell neoplasms such as lymphoma, PCT, and MCT. FSA and OSA were each identified in 6 cats (7%), lymphoma and PCT in 3 cats each (3%), chondrosarcoma and MCT in 2% of cases, and rare neoplasms including fibrolipoma, POF, and rhabdomyosarcoma were observed in 1 cat each (1%).

Clinical and demographic characteristics are summarized in Table 1. Cats with epithelial neoplasms (*N* = 67) were significantly older than those with mesenchymal neoplasms (*N* = 25), with mean ages of 11.0 ± 3.8 and 6.8 ± 4.1 years, respectively (*p* < 0.001). Body weight (3.9 ± 1.0 kg vs. 4.0 ± 1.2 kg, *p* = 0.714) and sex distribution (*p* = 0.295) did not differ significantly. Breed distribution differed (*p* = 0.027), with DSH cats more prevalent in the epithelial group (80.6%) than the mesenchymal group (56.0%). Less common breeds, including Bengal, Korat, and Scottish Fold, were observed only in the mesenchymal group. Clinical stage (*p* = 0.618), presence of dental disease (*p* = 0.315), and perioperative mortality (*p* = 0.669) did not differ significantly between groups.

**Figure 4:**
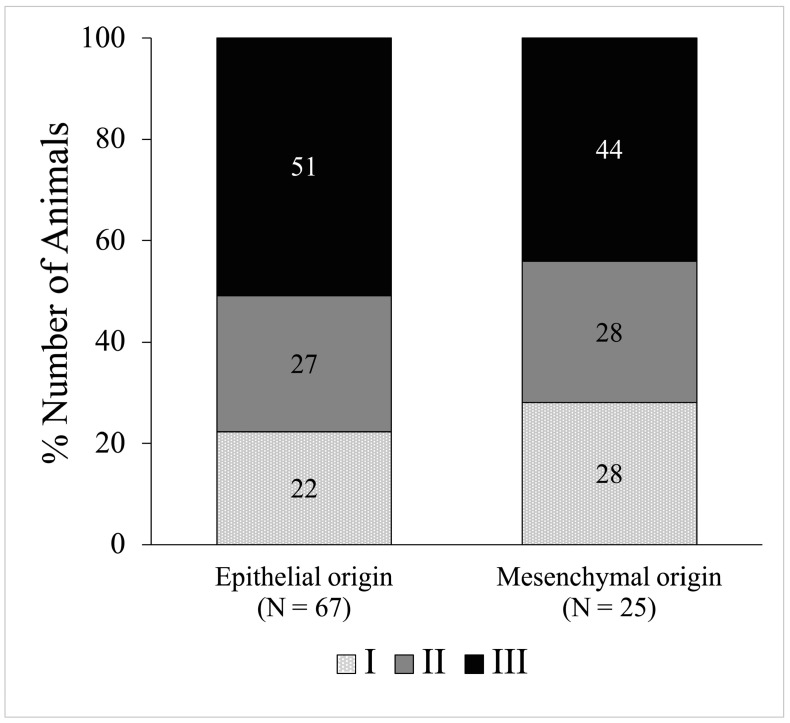
Comparison of feline oral neoplasm staging according to the WHO TNM system based on epithelial and mesenchymal origins. Among epithelial neoplasms, 22% of animals were classified as stage I, 27% as stage II, and 51% as stage III. In contrast, mesenchymal neoplasms showed 28% of animals in stage I, 28% in stage II, and 44% in stage III. No significant association was found between neoplasm origin and stage.

Neoplasm staging based on WHO TNM criteria is shown in Figure 4. Among epithelial neoplasms, 15 cases (22%) were stage I, 18 cases (27%) stage II, and 34 cases (51%) stage III. Mesenchymal neoplasms included 7 cases (28%) stage I, 7 cases (28%) stage II, and 11 cases (44%) stage III. Neoplasm origin was not significantly associated with clinical stage distribution.

Anatomical distribution by neoplasm origin is presented in Table 2. The maxillary gingiva was the most frequently affected site overall, accounting for 14 (15.2%) epithelial and 10 (10.9%) mesenchymal neoplasms. Epithelial neoplasms were more common in the buccal mucosa (18; 19.6%) and sublingual region (9; 9.8%) compared with mesenchymal tumors (5; 5.4% and 2; 2.2%, respectively). Mandibular gingival neoplasms included 13 (14.1%) epithelial and 6 (6.5%) mesenchymal cases. Less common sites, including the upper lip (8; 8.7%) and tonsil (3; 3.3%), were observed only in the epithelial group, whereas lower lip and palatine neoplasms (1; 1.1% each) occurred in both origins. Neoplasm site was not significantly associated with neoplasm origin (*p* = 0.127).

Medication use was analyzed by age group (Table 3). Antibiotic administration was more frequent in senior cats (78.0%) compared to mature/adult cats (52.4%) (*p* = 0.014). Steroid use (8.0% vs. 9.5%, *p* = 0.796), feeding type (*p* = 0.791), and presence of dental disease (54.0% vs. 59.5%, *p* = 0.675) did not differ. A trend toward advanced clinical stages in senior cats was noted but was not statistically significant (*p* = 0.087). Perioperative mortality was higher in senior cats (12.0%) than mature/adult cats (2.4%), though the difference was not significant (*p* = 0.121).

Neoplasm size and patient characteristics are summarized in Table 4. Sixteen neoplasms measured ≤2 cm, and 76 neoplasms were >2 cm. Senior cats (>10 years) were more prevalent in the larger neoplasm group (60.5%) than in the small neoplasm group (25.0%) (*p* = 0.013). Body condition score (*p* = 0.753) and sex (*p* = 0.785) did not differ between groups. Most small neoplasms (≤2 cm) were stage I (93.8%), while most larger neoplasms (>2 cm) were stage II or III (92.1%) (*p* < 0.001). Perioperative mortality did not differ significantly by neoplasm size (*p* = 0.348).

## 4. Discussion

In the present study, feline oral neoplasms were predominantly of epithelial origin, with SCC comprising the majority of cases. Among the 92 cats evaluated, SCC represented 61 cases (67% of all neoplasms) and constituted the majority of epithelial neoplasms, supporting its status as the most prevalent malignant oral neoplasm in cats. This distribution aligns with previous retrospective and epidemiologic studies that consistently report SCC as the most common feline malignant oral neoplasm [3,4,5,10,22,23,24]. The low prevalence of other epithelial neoplasm types emphasizes the need for careful histopathological assessment and, where appropriate, immunohistochemical confirmation to ensure accurate diagnosis. FSA was the most frequently identified mesenchymal neoplasm subtype in this cohort, consistent with prior reports describing FSA as the second most common oral neoplasm in cats [1,23]. Rare neoplasm types, including PCT, POF, MCT, and chondrosarcoma, were occasionally identified, consistent with previous retrospective studies [4]. Feline oral melanoma was rare, supporting prior findings of its low incidence [7,22,23]. In contrast, oral melanoma is among the most common malignant oral neoplasms in dogs [25]. Benign neoplasms were uncommon in this cohort, with POF and fibrolipoma each identified in a single case (1%). The predominance of malignant entities likely reflects the biological distribution of feline oral neoplasms and referral bias toward submission of clinically aggressive lesions for histopathologic evaluation.

Cats with epithelial neoplasms were significantly older than those with mesenchymal neoplasms. Senior cats were more frequently affected, highlighting age as a key demographic factor [2,4]. Similar age-related trends have been reported in SCC across multiple studies [22,26], potentially reflecting cumulative exposure to carcinogenic or inflammatory stimuli over time. Breed distribution varied between neoplasm origins, with DSH cats being overrepresented among epithelial neoplasms. This agrees with a previous report in which more than half of feline oral neoplasms occurred in DSH cats [11]. In our cohort, DSH cats were overrepresented among epithelial neoplasms, while less common breeds such as Bengal, Korat, and Scottish Fold were mainly associated with mesenchymal neoplasms. Similarly, European Shorthair cats were overrepresented among cases of SCC in European cohorts [3,4]. Overall, these findings support prior observations indicating no clear sex or breed predilection for SCC [26].

In addition to host-related factors, environmental influences have been suggested to contribute to SCC development, though definitive causal links remain unclear [12,13,14,15,16]. In tropical regions such as Thailand, increased sunlight exposure and outdoor housing may contribute to diseases of oral epithelial tissues. UV light has been linked to skin cancer development [17]. In the present cohort, epithelial neoplasms (73%) predominated over mesenchymal neoplasms (27%). This proportion is comparable to reports from Western countries, where epithelial neoplasms—particularly SCC—also predominate, rather than indicating a distinct geographic pattern. The mean age of cats with epithelial neoplasms (11.0 ± 3.8 years old) was likewise similar to that described in Western cohorts, in which SCC is typically diagnosed in senior cats. As no direct comparative cohort or exposure data were included, the influence of geographic or environmental factors cannot be determined from the present study and remains speculative.

Cats with chronic or comorbid conditions were more likely to receive antibiotic treatment, highlighting the influence of underlying health status on therapeutic decisions. Antibiotic use was significantly more frequent in senior cats (78.0%) than in mature or adult cats (52.4%; *p* = 0.014), likely reflecting increased clinical intervention for oral pain, inflammation, or secondary infection associated with advanced oral neoplasms and concurrent systemic conditions, rather than a causal role in tumor development. Similar patterns have been observed in previous large-scale analyses, where antibiotic use increased not only with age [27] but also in cats with systemic conditions such as endocrine disorders and retroviral infections [28], suggesting that comorbidity rather than age alone may drive prescribing patterns. Importantly, antibiotic administration in the present study was interpreted as a consequence of clinical signs prompting treatment, not as a risk factor for oral neoplasms. In contrast, steroid use, feeding type, and the presence of concurrent dental disease did not differ significantly between age groups, suggesting that these factors were not associated with the risk of oral neoplasms.

## 5. Limitations

**Figure 5:**
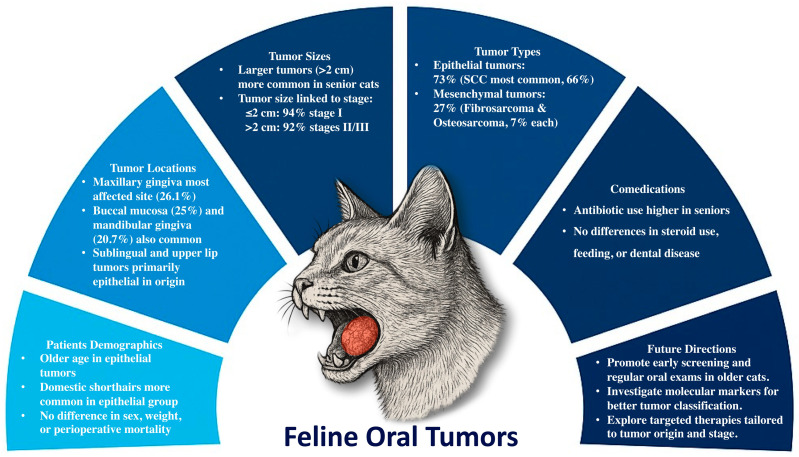
Summary of key findings in feline oral neoplasms. Neoplasms most frequently affected the maxillary gingiva, with larger lesions (>2 cm) associated with advanced clinical stages. Epithelial neoplasms, particularly SCC, were more common in older DSH cats. Antibiotic use was higher in senior cats, while sex, weight, and other clinical factors showed no significant differences. Future directions include encouraging early screening programs, identifying specific neoplasm markers for improved diagnosis, and developing targeted therapeutic approaches.

This study was retrospective and included cases from a single country, which may limit generalizability. In addition, all cases were derived from a single referral hospital, introducing potential single-center bias and limiting representation of the broader cat population in Thailand. Some medical records lacked complete clinical follow-up, and treatment outcomes beyond perioperative mortality were not available. Long-term outcome data, including disease-free interval and overall survival, were not assessed; therefore, the prognostic relevance of clinical staging could not be validated in this cohort. Environmental and dietary exposures were recorded in clinical histories but could not be quantitatively assessed, restricting evaluation of potential risk factors. Although histopathological evaluation remains essential for diagnosis [1,4,29], providing key information on neoplasm type, differentiation, and biological behavior, molecular data were not routinely available in this cohort. Molecular analyses are increasingly used to complement histopathology, improving understanding of neoplasm biology [30,31,32] and, in selected cases, supporting more precise classification [31]. However, such analyses were not performed in the present study. Furthermore, histologic grading for major tumor types, such as SCC and fibrosarcoma, was not consistently available, precluding assessment of grade as a prognostic factor. Figure 5 summarizes the main clinicopathologic patterns identified in feline oral tumors. In DSH cats, larger neoplasms were associated with more advanced stages, and SCC was most frequent in older animals. Antibiotic use was higher in senior cats, whereas sex, body weight, and dental disease prevalence did not differ by age. These findings support routine oral examinations for early detection [18].

## 6. Conclusions

Epithelial neoplasms, particularly SCC, accounted for two-thirds of feline oral neoplasms in this cohort. Mesenchymal neoplasms were less common but histologically diverse, with FSA and OSA most frequent. Cats with epithelial neoplasms were older than those with mesenchymal neoplasms; sex, body weight, and perioperative outcome did not differ significantly. Maxillary gingiva, buccal mucosa, and mandibular gingiva were the most commonly affected sites. Neoplasm size correlated strongly with clinical stage: small neoplasms (≤2 cm) were mostly stage I, while larger neoplasms (>2 cm) were stages II–III. Antibiotic use was higher among senior cats, potentially reflecting chronic inflammation or delayed detection. These results provide baseline epidemiological and clinicopathological data on feline oral neoplasms in Thailand, confirm the predominance of SCC, and describe the distribution of tumor size and clinical stage at presentation in this cohort.

## Figures and Tables

**Table 1:** Clinical and demographic characteristics of 92 cats with oral neoplasms by neoplasm origin. Cats with epithelial neoplasms (*N* = 67) were older than those with mesenchymal neoplasms (*N* = 25). Breed distribution differed between groups, with DSH cats more common in the epithelial group. No significant differences were found in body weight, sex, clinical stage, dental disease, or perioperative mortality.

Parameters	Epithelial Origin	Mesenchymal Origin	*p*-Value
No. of Patients	67	25	-
Age (years)	11.0 ± 3.8	6.8 ± 4.1	<0.001
Body Weight (kg)	3.9 ± 1.0	4.0 ± 1.2	0.714
Gender			
Male	32 (47.8%)	15 (60.0%)	0.295
Female	35 (52.2%)	10 (40.0%)	
Breed			
DSH	54 (80.6%)	14 (56.0%)	0.027
Persian	11 (16.4%)	7 (28.0%)	
Maine coon	1 (1.5%)	1 (4.0%)	
American wirehair	1 (1.5%)	0 (0.0%)	
Bengal	0 (0.0%)	1 (4.0%)	
Korat	0 (0.0%)	1 (4.0%)	
Scottish fold	0 (0.0%)	1 (4.0%)	
Clinical Stages			
I	14 (20.9%)	7 (28.0%)	0.618
II	16 (23.9%)	7 (28.0%)	
III	37 (55.2%)	11(44.0%)	
Concurrent Dental Diseases			
Yes	27 (40.3%)	13 (52.0%)	0.315
No	40 (59.7%)	12 (48.0%)	
Perioperative Mortality			
Yes	6 (9.0%)	1 (4.0%)	0.669
No	61 (91.0%)	24 (96.0%)	

**Table 2:** Distribution of oral neoplasm sites by neoplasm origin in 92 Cats. The maxillary gingiva was the most common neoplasm site, especially in mesenchymal cases. Buccal mucosa and sublingual neoplasms were more frequently epithelial, while mandibular gingiva neoplasms were evenly distributed. Less common sites, such as the upper lip and tonsil, were observed only in the epithelial group.

Neoplasm Site	Epithelial Origin N (%)	Mesenchymal Origin N (%)
Maxilla Gingival	14 (15.2%)	10 (10.9%)
Buccal Mucosal	18 (19.6%)	5 (5.4%)
Mandible Gingiva	13 (14.1%)	6 (6.5%)
Sublingual	9 (9.8%)	2 (2.2%)
Upper Lip	8 (8.7%)	0 (0.0%)
Tonsil	3 (3.3%)	0 (0.0%)
Lower Lip	1 (1.1%)	1 (1.1%)
Palatine	1 (1.1%)	1 (1.1%)

**Table 3:** Medication use and clinical features in senior vs. mature/adult cats with oral neoplasms. Prolonged antibiotic use was significantly more common in senior cats (78.0%) than in mature/adult cats (52.4%). No significant differences were observed between groups in steroid use, feeding type, dental disease, clinical stage, or perioperative mortality, though senior cats showed a trend toward more advanced disease and higher mortality.

Parameters	Senior Cats	Mature/Adult Cats	*p*-Value
No. of Patients	50	42	-
Prolong Antibiotic Use			
Yes	39 (78.0%)	22 (52.4%)	0.014
No	11 (22.0%)	20 (47.6%)	
Steroid Use			
Yes	4 (8.0%)	4 (9.5%)	0.796
No	46 (92.0%)	38 (90.5%)	
Feeding Type			
Ad libitum	24 (45.2%)	19 (48.0%)	0.791
Meal	26 (54.8%)	23 (52.0%)	
Dental Disease			
Yes	27 (54.0%)	25 (59.5%)	0.675
No	23 (46.0%)	17 (40.5%)	
Clinical Stages			
I	7 (14.0%)	14 (33.3%)	0.087
II	14 (28.0%)	9 (21.4%)	
III	29 (58.0%)	19 (45.2%)	
Perioperative Mortality			
Yes	6 (12.0%)	1 (2.4%)	0.121
No	44 (88.0%)	41 (97.6%)	

**Table 4:** Association between neoplasm size and patient parameters in cats with oral neoplasms. Among 92 cats, larger neoplasms (>2 cm) were significantly more common in senior cats and associated with more advanced clinical stages. No significant differences were found between neoplasm size groups in body condition, sex, or perioperative mortality.

Parameters	Diameter (≤2 cm)	Diameter (>2 cm)	*p*-Value
No. of Patients	16	76	
Senior cats (>10 years old)			
Yes	4 (25.0%)	46 (60.5%)	0.013
No	12 (75.0%)	30 (39.5%)	
Body Condition			
Underweight	7 (43.8%)	30 (39.5%)	0.753
Normal	9 (56.2%)	40 (52.6%)	
Obese	0	6 (7.9%)	
Gender			
Male	9 (56.2%)	38 (50.0%)	0.785
Female	7 (43.8%)	38 (50.0%)	
Clinical Stages			
I	15 (93.8%)	6 (7.9%)	<0.001
II	0 (0.0%)	23 (30.3%)	
III	1 (6.2%)	47 (61.8%)	
Perioperative Mortality			
Yes	0 (0.0%)	7 (9.2%)	0.348
No	16 (100.0%)	69 (90.8%)	

## Data Availability

The datasets generated or analyzed during this study are available from the corresponding author upon reasonable request.

## Abbreviations

The following abbreviations are used in this manuscript:

AAFP	American Association of Feline Practitioners
DSH	Domestic Shorthair
H&E	Hematoxylin and Eosin
OSA	Osteosarcoma
OSCC	Oral Squamous Cell Carcinoma
POF	Peripheral Odontogenic Fibroma
TNM	Tumor–Node–Metastasis
WHO	World Health Organization
